# Patient perceptions of barriers and facilitators for self-care in surgical fast-track programmes related to capability, opportunity and motivation: a theory-based qualitative study in Sweden

**DOI:** 10.1177/17449871261456609

**Published:** 2026-07-05

**Authors:** Pär Wennberg, Kerstin Eriksson, Margit Neher, Anders Broström, Anna Granath, Lotta Wikström

**Affiliations:** Research Leader, Research, Education, Development and Innovation Department, Skaraborg Hospital, Skövde, Sweden; School of Health and Welfare, Jönköping University, Jönköping, Sweden; Senior Lecturer, School of Health and Welfare, Jönköping University, Jönköping, Sweden; Department of Anaesthesia and Intensive Care, Ryhov County Hospital, Jönköping, Sweden; Senior Lecturer, School of Health and Welfare, Halmstad University, Halmstad, Sweden; Professor, Department of Nursing, School of Health and Welfare, Jönköping University, Jönköping, Sweden; Department of Clinical Neurophysiology, University Hospital, Linköping, Sweden; Lecturer, Nursing Department, School of Health Sciences, Jönköping University, Jönköping, Sweden; Senior Lecturer, Department of Nursing, School of Health and Welfare, Jönköping University, Jönköping, Sweden

**Keywords:** behaviour, motivation, perioperative care, recovery after surgery, self-care, social support

## Abstract

**Background:**

Surgical care increasingly shifts pre- and postoperative care responsibility to patient self-care at home. By using the capability, opportunity, motivation–behaviour (COM-B) framework, patient determinants influencing self-care can be explained.

**Aim:**

The aim was to describe patients’ experienced facilitators of and barriers to self-care in surgical fast-track programmes related to capability, opportunity and motivation.

**Methods:**

A qualitative design study with semi-structured interviews was conducted among 27 general and orthopaedic surgery patients at three Swedish hospitals. Data were analysed deductively using the COM-B framework.

**Results:**

The patients’ self-care experiences were explained by capability, opportunity and motivation and the dynamic interaction between these factors. A key analytical finding was the pivotal role of family and friends, whose emotional and practical support strengthened patients’ capability and motivation. Facilitators for behaviour change included prior surgical experience, clear information, physical ability, social and professional support, optimism and realistic goals. Barriers included cognitive and physical limitations, pain, fatigue and emotional distress.

**Conclusions:**

Findings highlight family involvement as an underused resource and support policy development of person centred pre- and postoperative self-care strategies. This study advances nursing theory by applying the COM-B model to illuminate interdependent behavioural processes in surgical self-care; themes could occasionally overlap.

## Background

Patient responsibility in the process of planned surgery has increased in the global rapid development of rapid surgeries due to proven safe care and cost-effectiveness. (Elsarrag et al., 2019; Joliat et al., 2020; Spanjersberg et al., 2011; Zhu et al., 2017). Fast-track surgeries mean that pre- and postoperative care, which earlier was provided by healthcare professionals, has been converted to self-care in the home environment for a large population that has undergone planned surgery (Omling et al., 2018). In Swedish healthcare, the definition of self-care from the legislation concerns a clinical context: ‘Self-care is when a patient is allowed to perform healthcare activities at home, either by themselves or with the help of a relative or a personal assistant’. This can, for example, involve medication or dressing a wound (Parliament, 2022). This means that healthcare professionals must make individual decisions regarding patients’ own and surrounding resources when making self-care recommendations.

Self-care aiming to optimise effects of surgical procedures include preoperative preparation routines, health-promoting recommendations and postoperative activities. Preoperatively, recommendations generally consist of cessation of alcohol and smoking, exercise, nutrition, hygiene activities and fasting (Gustafsson et al., 2019; Wainwright et al., 2020). Postoperatively, self-care activities are not as well defined as they vary between different surgical procedures, additionally patients’ varied needs in the recovery process are in many surgeries not well defined. A scoping review concerning surgical e-Health applications identified from healthcare scheduled activities/exercises, management of surgical wounds, intake of nutritious diets, pain management and monitoring of symptoms, with little regard for individualisation (Wikström et al., 2022). To help patients understand the importance of pre- and postoperative recommended self-care, thorough information, education and counselling are strongly recommended by the Enhanced Recovery After Surgery (ERAS) society (ERAS-Society, 2025). These activities reduce preoperative anxiety and help patients return home after surgery (Hounsome et al., 2017). In contrast, a lack of compliance can lead to cancelled surgery (Al Talalwah and McIltrot, 2019) and unnecessarily long recovery times (Buus et al., 2021). Studies of patients’ experiences of understanding self-care responsibilities reveal that healthcare information is sometimes too limited and sometimes too comprehensive (Buus et al., 2021). Additionally, patients experience several challenges in achieving compliance, such as troublesome symptoms, tiredness and forgetfulness (Aydin and Celik, 2024; Berg et al., 2019; Høvik et al., 2018; Sibbern et al., 2017; Thoen et al., 2024).

Research has provided some previous understanding of factors influencing compliance to recommendations for pre- and postoperative self-care. As noted by Larsson et al., healthcare professionals and patients’ families and friends have been shown to have an impact on patients’ experiences of safety in the perioperative process. In addition, in-depth patient information may facilitate compliance with recommendations while symptom burden may be a barrier (Larsson et al., 2023).

However, for individual patients, compliance to recommendations for self-care requires a reshaping of personal habits and behaviours, and there is a more recent recognition of the need for individualised support based on patients’ resources. This need is rarely addressed in current information or in E-health applications (Buus et al., 2021; Wikström et al., 2022).

The theoretical framework of the capability, opportunity, motivation–behaviour framework (COM-B) (Michie et al., 2011) was used, to our knowledge for the first time, to identify missing aspects of behaviour change in this context. COM-B has previously been used in other settings to understand self-care from the from the patient’s perspective as well as to understand facilitators and barriers to behaviour change (Odzakovic et al., 2025; Whittal et al., 2021; Zou et al., 2017). Using the theoretical domain framework (TDF) supports identifying COM-B-related components. The TDF has synthesised several theories into 14 domains, found in the subcomponents of COM-B (Atkins et al., 2016, 2017; Cane et al., 2012). These enable a detailed analysis of behavioural influences and a systematic identification of relevant TDF domains to locate modifiable factors that may contribute to variation in behaviour (Atkins et al., 2016; Odzakovic et al., 2025). By analysing which aspects of capability, opportunity and motivation are lacking or prominent, it becomes possible to understand why behaviour change succeeds or fails (Michie et al., 2011).

This study focused on patients’ experiences of behaviour changes related to pre- and postoperative self-care from a short perspective, that is, the first weeks after surgery. To our knowledge, experienced facilitators and barriers to behaviour change in this context have not been studied. As there is a limited understanding of the mechanisms behind individual patient behaviour change, this study takes a theory-based approach to identify missing aspects that need to be incorporated into designing adequate behaviour change support. Therefore, our aim was: to describe patients’ experienced facilitators of and barriers to self-care in surgical fast-track programmes related to capability, opportunity and motivation.

## Methods

### Design

This was a qualitative study based on semi-structured interviews with a deductive analysis, conducted with patients from three secondary-care hospitals in southern Sweden: one large regional facility, and two medium-sized district hospitals.

### Sampling and recruitment

The inclusion criteria were Swedish speaking, no significant cognitive impairment, adult patients who had undergone inpatient surgery at one of the three hospitals and spent at least one night at the hospital after surgery. To ensure a broad range of experiences a total of 27 participants were recruited with strategic sampling considering age, sex, hospital, surgical intervention (general surgery or orthopaedic) and previous surgical experience. Purposeful sampling based on predefined criteria was used to recruit participants with direct experience of the phenomenon, while also striving for variation. Participants were also recruited through intermediaries strategically but still convenience-based, which may have influenced who was informed about the study and thereby introduced potential recruitment bias.

Clinicians at the three participating hospitals identified patients on the basis of the inclusion criteria. Patients were then invited to participate prior to surgery in connection with their hospital admission. Those who agreed to participate received both oral and written information. One to two weeks after the surgery, the interviewer contacted the patient by phone to schedule an interview.

### Data collection

Data collection was conducted from April 2021 to October 2022. The semi-structured interview guide was developed by senior researchers with experience and expertise in qualitative research on the basis of the COM-B theoretical framework (Supplemental File 1). A theoretical matrix was constructed to assist in the development of the interview guide (Supplemental File 2). Three researchers/nurses trained in qualitative methods, conducted the interviews and each interview lasted between 23 and 63 minutes, with a median of 41 minutes. The participants were asked about their experiences regarding their situation and self-care in connection with the surgery. All interviews were conducted by telephone for practical and geographical reasons; telephone interviewing is recognised as a suitable method for eliciting personal experiences in healthcare research (Musselwhite et al., 2007). Interviews were audio-recorded and transcribed verbatim.

### Data analysis

**Figure 1.**
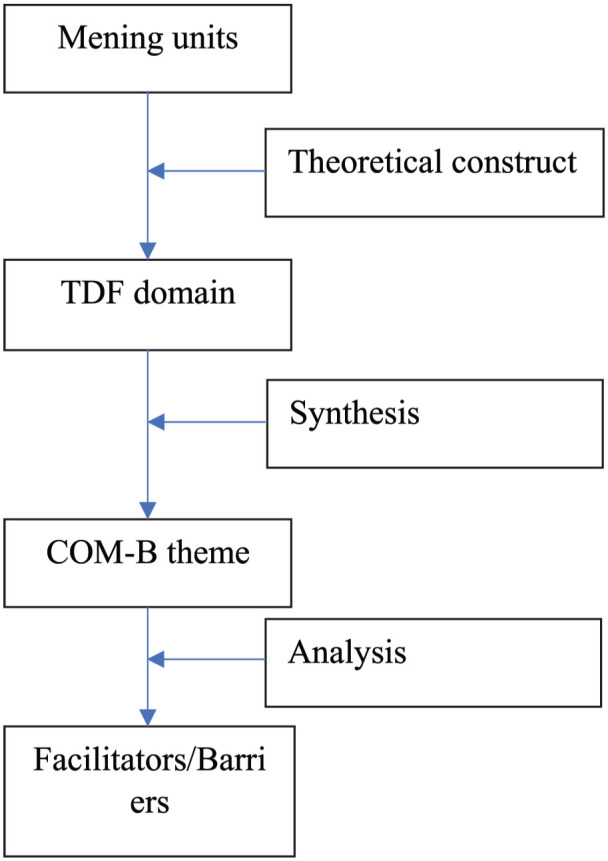
Visualisation of the deductive analytic process.

A deductive, semantic, thematic analysis inspired by Braun and Clarke (2006) was undertaken, applying predefined theoretical categories from the TDF and subsequently the COM-B model. The analytical process is visualised in Figure 1. The transcripts were first read repeatedly to ensure familiarisation. Meaning units that described patients’ experiences of self-care in relation to their surgery were identified across all interviews. Each meaning unit was condensed and then deductively assigned to the most relevant TDF domain. The theoretical matrix, including TDF domains and their associated theoretical constructs, guided the coding into the TDF domain that best captured the behavioural determinant reflected in each meaning unit. The first three transcripts were coded independently by three researchers and then discussed together to establish a shared understanding of how meaning units should be assigned within the deductive coding framework. Next, the content within each TDF domains was synthesised and interpreted through the six themes provided by the COM-B framework. This step reflected the structural relationship in which TDF domains map onto COM-B themes. The COM-B model thereby helped explain how clusters of TDF coded meaning units related to behavioural mechanisms. Within each COM-B theme, the interpreted material was analysed to determine whether the described experiences of functioned as facilitators or barriers to postoperative self-care behaviour (Table 3). This step enabled an explanatory understanding of how specific aspects of capability, opportunity and motivation supported or hindered behaviour change. Meaning units, TDF domains, COM-B synthesis and facilitator/barrier classifications were repeatedly compared back to the transcripts to ensure confirmability. Representative quotations were selected to illustrate each COM-B theme and its corresponding facilitators and barriers. Facilitators and barriers were finally abstracted into the summarising Table 2. Here, the semantic approach helped us to stay close to participants’ descriptions. NVivo 14 software (Lumivero)supported data organisation throughout the coding process, after sorting meaning units to TDF domains and COM-B themes, data were exported to excel to get an overview of the data which assisted the synthesis.

Code saturation – no new TDF domain related codes – was reached after 15 interviews, whereas meaning saturation – no new insights within existing COM-B categories – was reached after 25 interviews. We used a deductive thematic analysis guided by the COM-B framework as the aim of the study was to describe participants’ experiences, but we also wanted to understand these experiences in relation to established determinants of behaviour. Here, we wanted to work with an explanatory rather than purely exploratory approach. Using COM-B for analysis, enabled us to systematically examine capability-, opportunity- and motivation-related factors relevant to behaviour. An analysis example is provided in Table 1.

**Table 1.** An example of how the analysis was executed.

Meaning unit	Condensed meaning unit	Theoretical construct	TDF domain	COM-B theme	Facilitator/barrier
‘Yes, it contained general information, including contact numbers and things like that, and instructions on how to proceed, for example if you were taking any medications and that you should write them down and bring them with you, as well as some general preparations. But I’ve had surgery on that shoulder so many times now that I already knew most of it by heart anyway’. Patient 6	The experience from the previous surgery made the person feel confident about what was going to happen	Knowledge	Knowledge	Psychological Capability	Facilitator: Having knowledge from previous experiences with surgery and healthcare

COM-B: capability, opportunity, motivation–behaviour; TDF: theoretical domain framework.

### Trustworthiness

Credibility was supported through iterative engagement with the data and reflexive discussions within the research team. Quotations were used to illustrate the analytic claims and ensure transparency between the data and the themes. Dependability was strengthened through an audit trail documenting analytic decisions, including reflexive memos, meeting notes and versioned coding files. Confirmability was ensured through reflexive awareness of theoretical preconceptions related to the COM-B framework, that is, the matrix was kept at close hand during all analysis work. Team-based discussions challenged individual assumptions, and data units that did not align with COM-B were retained and considered to avoid forcing interpretations. Transferability was supported by providing descriptions of the study context, participant characteristics and recruitment process so that readers may judge the applicability of the findings to similar clinical settings.

### Ethical considerations

This study received ethical approval from the Swedish Ethical Review Authority and adhered to ethical guidelines in accordance with the Declaration of Helsinki (World Medical Association, 2024). All participants received written information about the study’s purpose, the voluntary nature of participation and their right to withdraw at any time without consequences. Informed consent was obtained prior to the interviews. To ensure confidentiality, all collected material was anonymised, and the data were stored securely. This study has been reported in line with the COREQ guidelines (Tong et al., 2007) – see Supplemental File 3 for the checklist.

## Results

A total of 27 patients were interviewed, including 15 women and 12 men. Among them, 19 patients had undergone orthopaedic surgery, and 8 patients had undergone general surgery across three different hospitals. The demographic characteristics of the participants are described in Table 2.

**Table 2.** Demographic description of the included patients (*n* = 27).

Variable	*n* (%)
Sex	
Female	15 (56)
Age (years)	
18–30	2 (7)
31–50	8 (30)
51–70	11 (41)
>70	7 (26)
Comorbidity	10 (37)
Type of surgery	
Orthopaedic	19 (70)
General	8 (30)
Hospital	
A	13 (48)
Orthopaedic surgery	9(69)
B	8 (30)
Orthopaedic surgery	8(100)
C	6 (22)
Orthopaedic surgery	2(33)
Previous experience from surgery	23 (85)
Swedish origin	27 (100)

Hospitals A, B and C refer to distribution of study participants at the three different hospitals where the participating patients had undergone surgery.

The patients’ experiences with self-care in the results are reflected by facilitators and barriers to carrying out self-care and are categorised according to the COM-B framework (Table 3).

**Table 3.** Facilitators and barriers in self-care connected to surgery.

COM-B domain*	Facilitators	Barriers
Psychological capability	Having the ability to acquire self-care information Having knowledge from previous experiences with surgery and healthcare Being able to overcome information gaps by improvising Being able to adapt to new activities and routines	Feeling cognitive impaired with limited memory and understanding instructions Having limited knowledge about surgical complications
Physical capability	Having physical capability to carry out selfcare tasks	Having limited skills/ability to relieve pain Being restricted due to disturbed sleep and surgical fatigue
Social opportunity	Having practical assistance from family and friends Having emotional support from family and friends Having family and friends present during discharge discussions	Having the need to have contacts with several different healthcare providers
Physical opportunity	Having access to professional support from healthcare with preparations of the home environment before surgery Having practical support from family and friends assisting with self-care tasks Having access to aids postoperatively	Having limited knowledge and understanding of how to use aids Having experience from limited healthcare opening hours Experiencing general and not personalised information
Reflective motivation	Being optimistic about the future Having forward-looking personal goals Feeling confident from previous personal experience from healthcare/surgeries Having emotional support from family and friends as well as healthcare Having access to individualised support before discharge	Having worries about when to contact healthcare Having unrealistic expectations from healthcare
Automatic motivation	Feeling positive emotional support from family and friends as well as healthcare	Having emotional challenges

* Capability refers to the individual’s psychological and physical capability to engage, and opportunity is defined as the physical and social factors lying outside the individual. In this context, physical opportunities can be described as healthcare resources, whereas social opportunities can be existing roles and norms within society. Reflective motivation includes beliefs in oneself, optimism, intentions and goals, whereas automatic motivation includes reinforcement and emotions.

### Capability

Postoperative self-care is a dynamic process influenced by multiple factors that impact the ability to perform it. Self-care information and prior surgical experience had a positive impact on patients’ confidence in managing self-care and support from family and friends was also important. However, factors such as uncertainty, pain, cognitive overload, limited physical function and fatigue could create significant barriers to successfully follow self-care recommendations.

#### Psychological capability

*Facilitators in* psychological capability covers knowledge and cognitive skills which are reflected by the ability to remember, concentrate and make decisions and are critical for a positive recovery experience. Patients highlighted the importance of self-care information given preoperatively to promote their knowledge of how to perform self-care as expected from healthcare. Patients who received information about expected postoperative side effects, including guidance on when to seek medical attention and patients with previous experiences with surgery and healthcare settings, reported a greater understanding and thereby a greater sense of control in their recovery.

When I started to feel it [an expected side effect], I immediately understood what it was and that felt reassuring. (Patient 26)

Having gone through it before [having experience from surgery] is reassuring as well. (Patient 27)

The ability to adapt to new activities and routines positively impacted self-care. Those receiving encouragement or receiving advice for self-care from healthcare or family and friends reported feeling safe. Patients who received clear and structured instructions before surgery as well as prior to discharge often found it easier to follow self-care routines.

It [the preoperative instructions] outlined what I should do and so on and this with showering and changing the sheets and all that so I did that so it was, it was clearly stated there that yes, what you should do and not do. (Patient 24)

Improvising was a cognitive skill that patients used to overcome information gaps. Patients who were not informed about the needs for preparing their home environment could facilitate their recovery on their own. For example, placing a stool in the shower to allow seated bathing or rearranging their furniture to facilitate physical activities.

Patients lacking clear information reported feelings of uncertainty and anxiety, perceiving these as barriers to psychological capability. Patients with self-reported cognitive impairment such as difficulties with memory and sometimes understanding instructions reported difficulties in deciding when to act on self-care matters such as pain relief and wound care. These patients also reported that contact with healthcare for advice could be a barrier. They avoided contacting the hospital and instead sought support, from familiar contacts such as the local pharmacy or family and friends. Issues demanding medical knowledge in the decision-making processes, such as adjusting pain management on their own support, were challenging without support from healthcare.

I can’t handle morphine . . . so I had to call them to make a lot of changes. (Patient 11)

Patients who experienced complications after surgery expressed a preference for returning to the hospital ward instead of dealing with complications, they had no knowledge of, such as problems with wound healing, from home.

I would have preferred to go back to the ward [to handle wound complications]. (Patient 14)

#### Physical capability

Patients facing physical barriers such as pain and fatigue experienced challenging recovery as physical capability plays a central role where strength, endurance and mobility directly impact patients’ ability to perform self-care and achieving postoperative recovery.

Facilitators in physical capability included structured information and practical guidance to facilitated self-care. A clear tapering schedule for pain-relieving medication provided by healthcare, helped patients understand the importance of following their scheduled dosages, reducing the risk of adverse effects and misuse.

This time, I got a tapering schedule, which I never had before. (Patient 5)

Barriers in physical capability was explained by limited physical capacity combined with insufficient aids and guidance from healthcare providers made some patients feel constrained and alone in their self-care. Patients explained how pain hindered self-care by restricting their physical ability and thereby becoming a barrier to performing recommended exercises.

I've been in a lot of pain, so now at the beginning here now, so that for the most part I've only been able to lie down in bed. (Patient 4)

Barriers to behavioural regulation and physical adaptation included disruptions to sleep patterns and limitations in activities due to the surgery. Patients described difficulties in finding a comfortable sleeping position postoperatively, which increased their fatigue in the daytime. Physical challenges, such as needing more rest due to the fatigue caused by the surgery, could also act as barriers, but not all patients had information about this.

But I probably really needed a little more [information] so that someone explained [that it takes time to go back to normal daily activities], maybe I can’t really go back to normal those first few days. (Patient 26)

### Opportunity

Facilitating resources for successful factors lying outside patients’ personal capacity include support from family and friends and access to aids adapted to the type of surgery, whereas insufficient healthcare resources when needed pose significant barriers to safe and independent recovery.

#### Social opportunity

Social support from family and friends was an important facilitator in social opportunity for patients’ ability to manage self-care. Their support provided both practical and emotional assistance. Practical assistance, such as cooking, cleaning and medication, was essential for managing daily household chores and enabled focus on self-care activities.

His [the patient’s son] grandmother came to pick him up. . . that made things a lot easier. (Patient 4)

Having family and friends present during discharge discussions was valuable, as it assisted in the retention of instructions and provided support in understanding the information.

We had double ears listening and able to ask questions, which was helpful. (Patient 7)

Patients not living with a life partner managed their self-care on their own. At the same time, they also realised that having practical and emotional assistance from family and friends could have contributed to support during the recovery process.

With my life situation as it is, I'm used to being by myself a lot, but of course it might have been nice to have a partner or similar [in situations of self-care connected to surgery]. (Patient 24)

The surgical process means that patients have to be in contact with multiple institutions, such as primary care healthcare professionals, hospital healthcare professionals and rehabilitation services. Patients see this as a barrier to self-care as not every institution has the knowledge of the specific surgery. To feel safe, patients therefore prefer a single, continuous point of contact throughout the entire process, ideally within the surgical unit.

I would rather have seen that from the day the primary care doctor sends the referral, and you get to go to the hospital, I think the hospital should take over from there, not until they have operated on you. I think they should handle the whole part because they are the most competent. They have done the surgeries, they know the medication, they know everything. (Patient 14)

#### Physical opportunity

Information about preoperative preparations were generally easy to understand. Family and friends contributed to correct surgical preparedness by assisting with preoperative self-care tasks, such as assisting with the preoperative shower, which alleviated patients’ worries about doing something wrong. Professional support from healthcare providers in preparation of the home environment before surgery contributed positively to patients’ ability to manage their self-care after surgery. Access to necessary aids adapted to the type of surgery and recommendations for a prepared home environment supported patients in regaining mobility in a safe manner. Family and friends also contributed to this physical preparedness by assisting with preoperative self-care tasks. Postoperatively, patients emphasised the importance of the right aids in place, such as walkers and shower chairs, which enabled them to perform daily activities independently.

Yes, the walker is good . . . it is a security for me and my wife when I walk around. (Patient 7)

Barriers in physical opportunity could be explained by patients that had not gained an understanding of how to use aids such as crutches, which complicated daily activities.

It’s difficult when you’re always holding something, and your hands aren’t free [using crutches]. (Patient 22)

Additionally, limited healthcare opening hours, particularly during holiday seasons, add stress and uncertainty, disrupting rehabilitation continuity.

They [the medical rehabilitation facilities] limit their hours and mess around. (Patient 5)

### Motivation

Reflective motivation is reflected in the individuals’ beliefs, goals and confidence in their capabilities, shaping their engagement in self-care. Patients’ motivation for self-care is strongly influenced by the combination of earlier experiences, clear personal goals of regaining health and support from family and friends. Additionally, supportive healthcare structures, where precise information and emotional support are key facilitators, are needed. Inadequate information, unrealistic expectations and the absence of continuous feedback from healthcare providers in the planning of care could create anxiety and diminish patients’ engagement in their recovery.

#### Reflective motivation

Optimism and a forward-looking perspective where personal goals were set emerged as strong facilitators of recovery. Patients reported a determined desire to achieve a better state of health.

This is my life’s chance to get rid of this pain. (Patient 6)

Patients’ previous experiences, particularly in healthcare, along with emotional support from family and friends as well as healthcare, often reinforced their motivation for active participation in self-care. Patients felt confident when they recognised the similarities of healthcare pre- and postoperative routines for different surgical episodes.

I knew what was going to happen so in that way I kind of felt that it was just ‘go ahead’ so I took hold of my things where I felt I needed to fix things and so on, but it's like I said before that a lot is from my own experience. (Patient 4)

Confidence in one’s own capabilities was facilitated by the availability of individualised support and information before discharge.

Yes, I did the pain assessment myself when I felt that it was so good that I could go home because there were no problems to stay for one or two more days if I wanted to [the patient had a choice to stay at the hospital]. (Patient 5)

The absence of individualised rehabilitation plans could create barriers, allowing patients to feel less capable in managing their care at home. A lack of specific postoperative information on complications or when to contact healthcare could create uncertainty and act as a barrier to self-care.

No, nobody mentioned what could happen [after surgery], here or there [did not know what to do. . .]. (Patient 10)

Unrealistic expectations from healthcare providers regarding preoperative preparations (generalised recommendations) led to feelings of disappointment that challenged the motivation for self-care, such as the difficulty of achieving rapid weight loss preoperatively.

You can’t lose weight in a month when you haven’t been able to do it before. (Patient 14)

#### Automatic motivation

Automatic motivation includes reinforcement and emotions. To overcome fears in self-care patients needed individually adapted reinforcements from the healthcare professionals. Support from family and friends were important when patients were experiencing mood fluctuations during the recovery to keep up motivation to undertake self-care activities.

Continuous positive reinforcement of information from healthcare providers and family and friends was critical and a facilitator for sustaining self-care motivation. Fear of postoperative complications, such as infections, also reinforced adherence to self-care routines. Emotional support from healthcare professionals at the hospital provided reassurance and motivated patients to overcome their fears, such as administering their blood clot prevention injections.

I did it [the injection] myself, with support from the staff, the first day at the hospital, since I have a bit of a fear of needles, it’s a real source of stress for me to have to do it every day, but I have grown into it. (Patient 3)

Emotional support offered a sense of security in managing self-care. Additionally, emotional encouragement from family and friends offered motivation, making self-care tasks more manageable.

It gives both understanding and someone [the wife] who can push you a little when needed. (Patient 7)

Emotional challenges, such as mood fluctuations, could act as barriers to self-care. Patients are not able to organise their thoughts when they feel sad and low in energy.

## Discussion

This study is the first to explore self-care facilitators and barriers focusing on a behavioural perspective in surgical care. Findings highlight the multifaceted nature of pre- and postoperative self-care following fast-track inpatient surgery, emphasising the relationship between factors facilitating or hindering patients to undertake expected self-care behaviour change. The behaviour change is needed from the patient and closely connected to carrying out self-care to gain recovery. The relationship between capability, opportunity and motivation in behaviour change determines whether patients perceive self-care as manageable (facilitators) or overwhelming (barriers). The following aspects from the COM-B framework are important in the conditions that the healthcare system needs to consider when delegating activities to self-care in connection with inpatient surgery.

Psychological capability, encompassing knowledge, cognitive skills and emotional preparedness, played a central role in patients’ ability to manage self-care. The findings indicate that psychological capabilities varied depending on the cognitive ability to process and request information, as well as the ability to adapt to new activities and routines. Consistent with earlier research (Buus et al., 2021; Høvik et al., 2018), those who received structured and comprehensive preoperative information reported a greater sense of security, facilitating self-care. Furthermore, previous experiences of surgery instilled positive recognition and enhanced confidence and adaptability, facilitating understanding of the required behavioural changes. This aligns with studies demonstrating that familiarity with healthcare procedures supports patient engagement in care (Blöndal et al., 2022). In contrast, unclear or inadequate information contributed to uncertainty and anxiety, with some patients expressing a preference for hospitalisation over self-care at home. These findings underscore the importance of individualised information that accounts for health literacy and prior experiences. Low health literacy is known to correlate with poorer surgical outcomes (Aydin and Celik, 2024; Trutner et al., 2023). This underscores the importance of comprehensive and understandable information pre-surgery according to the findings in this study.

Physical capability influenced patients’ ability to engage in self-care and varied in relation to symptom burden, including pain, fatigue, weakness and sleep disturbances. These symptoms hindered patients’ ability to carry out self-care activities, confirming prior research identifying postoperative pain, fatigue and inadequate aids as barriers to recovery (Berg et al., 2019; Vikan et al., 2025). Sleep disturbances further delayed recovery, consistent with evidence linking disrupted rest to postoperative complications (Rampes et al., 2019). Physical capability was also shaped by the type and extent of the surgical procedure. Adequate baseline physical capacity appeared necessary to perform recommended postoperative training. When such prerequisites were limited, greater behavioural adaptation was required in other components of the COM-B model, particularly in terms of motivation and psychological capability and social themes. Addressing physical limitations through targeted informational and supportive interventions – such as individualised pain management plans and sleep hygiene recommendations – may help mitigate these barriers. Overall, these findings suggest that themes like physical capability are difficult to explain in isolation. Rather, the components: capability, opportunity and motivation interact dynamically; limitations in one domain (for example capability) necessitate compensatory resources in others. Behaviour changes to achieve postoperative self-care thus emerges from the interdependence of capability, opportunity and motivation and cannot be fully understood through discrete thematic categories.

Social opportunities meant that the presence of both practical and emotional support from family and friends enhanced self-care, while having to be responsible for multiple appointments in different places was experienced as an obstacle. The impact of family and friends as a facilitator to self-care was present in all interviews, even with participants living on their own. The high level of the importance of family and friends was rather surprising to us – and COM-B does not really provide the framework to reflect this fully across all themes. Patients with social support reported confidence and independence, consistent with previous research (Larsson et al., 2023). Practical assistance – such as help with household tasks – reduced stress and enabled focus on self-care, while emotional encouragement strengthened motivation for self-care. These findings reinforce evidence showing that social support enhances well-being and recovery (Orlas et al., 2021; Sibbern et al., 2017). Therefore, involving family and friends in self-care programmes is vital (Shen et al., 2025). However, even with strong social supportive networks, inadequate pain management remained a barrier to physical recovery. Since pain is a major barrier to postoperative recovery (Eriksson et al., 2020) and often poorly addressed in patient information, nursing interventions should prioritise personalised pain management strategies including non-pharmacological approaches (Blöndal et al., 2022).

Physical opportunities meant receiving support in facilitating changes to the home environment before surgery and having access to individualised support before going home.

Patients with preoperative home preparation experienced reduced obstacles in daily activities and increased adherence to self-care routines, which has been reported in previous research (Causey-Upton and Howell, 2017; Høvik et al., 2018). Additionally, family and friends contributed to this readiness, emphasising the interconnection between social and physical support. However, lack of familiarity with aid devices and healthcare resource limitations posed challenges. Limited access to healthcare, particularly during holidays, further delayed recovery. Ensuring consistent access to medical guidance and rehabilitation is therefore important to sustain recovery momentum.

Reflective motivation was shaped by prior experience, expectations, optimism and belief in recovery. Positive prior surgical experiences facilitated behavioural changes and demonstrated greater confidence in performing self-care routines, also shown in previous research (Blöndal et al., 2022). For patients without such experience, detailed and individualised preoperative information was essential to build perceived capability, whereas insufficient or generalised guidance increased uncertainty and anxiety. Goal-oriented thinking and an optimistic outlook strengthened engagement in self-care, consistent with evidence that goal setting enhances adherence (Roberts et al., 2021). However, unrealistic expectations, such as rapid preoperative weight loss or assuming responsibility for coordinating ongoing healthcare contacts, reduced motivation and led to frustration. These findings underscore the importance of realistic, individualised goal setting adapted to health literacy levels (Høvik et al., 2018; Paterick et al., 2017) and preferences for involvement (Vogel et al., 2023).

Automatic motivation, driven by emotion and habit, also influenced self-care behaviours. Fear of complications, particularly infection, promoted adherence to wound care routines, aligning with evidence that perceived health threats enhance compliance (Shelton et al., 2020). Yet excessive anxiety risked counterproductive effects, including avoidance and heightened stress (Lanini et al., 2022). Emotional support from family, friends and healthcare professionals reinforced motivation, helping patients manage procedural anxieties such as self-injection. Encouragement and clear communication from healthcare staff strengthened confidence and sustained engagement in self-care, highlighting the importance of empathetic, supportive nursing practice (Hesselink et al., 2020).

The findings emphasise that self-care following surgery is a behavioural process influenced by interacting psychological, physical and social dimensions. Applying the COM-B framework in perioperative nursing provides theoretical clarity and a structured approach to identifying behavioural determinants of self-care. Nurses are ideally placed to strengthen patients’ capability, opportunity and motivation through education, counselling and coordination of follow-up services. From a health and social care policy perspective, postoperative outcomes depend not only on surgical success but also on the resources and support patients’ access. Policy frameworks should thus extend responsibility for care beyond hospital boundaries, integrating community-based follow-up, caregiver education and digital tools to maintain continuity. Developing standardised, person-centred self-care strategies, co-designed with patients, families and friends could improve equity and efficiency across surgical services.

### Strengths and limitations

Most participants had prior experience with surgical procedures. This means that the findings may reflect the experiences of these patients more than those without such experience, potentially affecting the study’s credibility across all surgical patients. On the other hand, many of the experiences described by the patients were interpreted as generic and were well explained within the theoretical framework used. Additionally, the findings are largely supported by previous research, which can be considered to strengthen the study’s credibility. Conducting interviews by telephone may have influenced how the participants responded (i.e. risk of withholding information), potentially affecting the dependability of the study. The theme that appeared to be the most difficult for participants to elaborate on was ‘Barriers in automatic motivation’, which could be partially explained by the use of telephone interviews. That said, the interviews contained rich data, with a median duration of 41 minutes, suggesting that the participants did not intentionally withhold information. The COM-B theoretical framework was considered to work well as an explanatory tool for mapping self-care in fast-track surgery. Still, we found some challenges along the way. The coding of meaning units into TDF domains were sometimes challenging, as overlap between COM-B themes were present in some meaning units. The nature of the COM-B model and themes sometimes tended to be close in content, but there were still differences to be observed. This means that overlapping meaning units may have resulted in themes being lost in translation. Another limitation is that the complexity of behaviour change is not easily explained through separate themes as all themes are closely linked together. This is not a limitation in the COM-B framework but something that happens when, trying to explain a phenomenon through detailed analytical decomposition, one risks losing sight of the phenomenon as a whole. Yet, when taking limitations into consideration, we found the COM-B framework to be a valuable tool in our analysis.

## Implications

Perioperative teams and nursing practice should strengthen behaviour‑change support by individualising self‑care guidance, actively involving family and friends and ensuring continuity of information across settings. This means providing clear, structured and individualised information tailored to patients’ cognitive and physical abilities and the expected support from family and friends both before surgery and before discharge. Preoperative preparation of the home environment and access to appropriate aids should be coordinated and explained by healthcare to promote safety and independence. Finally, healthcare providers play a key role in offering ongoing support and follow-up through planned contacts or digital platforms to strengthen patient motivation, compliance with self-care to achieve recovery. The findings imply that nursing education should place emphasis on behaviour-change theory (such as the COM-B framework) to help nurses assess and tailor patient education that supports psychological, physical and motivational aspects of pre- and postoperative self-care. Further research should explore how family and friends, digital interventions, peer networks and community partnerships can enhance motivation and enablement for self-care. Longitudinal designs could track behavioural changes along the entire recovery trajectory.

## Conclusions

This study highlights that postoperative self-care is a complex and dynamic process, and effective pre- and postoperative self-care in acute care processes requires behavioural change. Patients’ ability to implement this change is strongly shaped by their cognitive ability to request and process information, their acquired health knowledge and motivation. This indicates that individual ability, opportunities and motivation are not only constituted by patients' own resources but also by the support available within the care system, which is one of the more important findings in this study. The study also shows that family and friends, often participate in and support the self-care process and are thus part of the expected behavioural changes. They therefore also need improved health knowledge to effectively support the behavioural demands placed on the patient – an aspect that has previously been overlooked or underestimated in acute care. Furthermore, described gaps in standardised information in acute care processes lead to patients seeking informal support from relatives, neighbours or community services, indicating that support for behavioural change in this context extends beyond the hospital system. Motivation was strongly influenced by emotional and practical support from social networks, previous experiences, realistic expectations, optimism and individualised recovery plans, whereas insufficient guidance and emotional challenges reduced engagement. Overall, enabling self-care after surgery requires a person-centred approach that integrates informational, emotional and practical support across the entire continuum of care. These findings suggest that healthcare professionals may need to look beyond conventional strategies to effectively promote behavioural change towards increased individual responsibility for postoperative self-care.

**Key points for policy, practice and research**

• Patients’ self-care behaviours before and after surgery depends on cognitive and physical capability, opportunity within the social and healthcare environment, and motivation shaped by prior experience and expectations.

• Clear, repeated, and tailored information enhances patients’ understanding and confidence, while symptom burden, and poor communication undermine self-care.

• Family, friends, and timely professional support are critical facilitators of self-care; lack of access and fragmented care are major barriers.

• A person-centred approach that integrates informational, emotional, and practical support strengthens motivation and self-management after surgery.

• The study contributes to nursing theory by applying the COM-B model to explain behavioural mechanisms in surgical self-care and informs policy through recommendations to implement structured, equitable self-care pathways within surgical programmes.

## Supplemental Material

sj-docx-1-jrn-10.1177_17449871261456609 – Supplemental material for Patient perceptions of barriers and facilitators for self-care in surgical fast-track programmes related to capability, opportunity and motivation: a theory-based qualitative study in Sweden Supplemental material, sj-docx-1-jrn-10.1177_17449871261456609 for Patient perceptions of barriers and facilitators for self-care in surgical fast-track programmes related to capability, opportunity and motivation: a theory-based qualitative study in Sweden by Pär Wennberg, Kerstin Eriksson, Margit Neher, Anders Broström, Anna Granath and Lotta Wikström in Journal of Research in Nursing
