# Supplementary material for: Patient perceptions of barriers and facilitators for self-care in surgical fast-track programmes related to capability, opportunity and motivation: a theory-based qualitative study in Sweden
Source: J Res Nurs. 2026 Jul 5:17449871261456609. Online ahead of print. doi: 10.1177/17449871261456609 (PMC13337552; doi:10.1177/17449871261456609)
Supplement: sj-docx-1-jrn-10.1177_17449871261456609 – Supplemental material for Patient perceptions of barriers and facilitators for self-care in surgical fast-track programmes related to capability, opportunity and motivation: a theory-based qualitative study in Sweden [file sj-docx-1-jrn-10.1177_17449871261456609.docx]

Supplementary file 1 COM-B

Interview guide

Introduction
The interview begins with a few general questions, followed by questions regarding your preparations for surgery. After that, we will ask about your self-care at home after surgery.

Definition of Self-Care
Self-care refers to the care you can perform independently. Examples include brushing your teeth, exercising, resting, taking prescribed medications, following recommended rehabilitation exercises, or seeking help when unable to manage a health issue on your own.

General questions

1. When you have questions or concerns about your health, can you describe how do you typically respond or act?

- How do you usually seek or obtain information about self-care or healthcare?
- When you need to discuss your health with someone, how do you usually communicate with healthcare services?

1. What factors influence your choice of method for seeking self-care information?

- Can you describe how these methods typically work for you?

1. What other ways of communicating with healthcare services are you aware of?

- What are your thoughts on these methods?

1. What emotions arise when you need to communicate with healthcare services?

- Do you experience any of the following:
- A sense of obligation? Anxiety? Stress? Security?
- A feeling of participation and consideration of your personal needs?

### **Before your surgery**

1. **Can you describe your experiences of communicating with healthcare services to obtain information before your surgery?**
   - How was the information provided? **Written information** from your clinic, **verbal information** from your clinic (in-person, phone, healthcare visit, video consultation), **E-health solutions**: internet, mobile applications
   - To what extent did the information accommodate your personal needs and preferences?
   - How accessible, readable, and useful was the information for you?
   - What were the advantages and disadvantages for you personally?
2. **Can you describe how the information helped you prepare for your surgery?**
   - Which aspects of the preparation process do you particularly remember?
   - How easy or difficult was it for you to understand the information about the necessary preparations?
   - What factors made your practical preparations easier or more challenging?

(If needed, remind the participant about common preparations, such as:)

- - **Fasting** (restrictions on food and drink)
  - **Hygiene** (showering before surgery)
  - **Daily activities/exercises/training**
  - **Smoking and alcohol cessation**
  - **Taking prescribed medications correctly**
  - **Packing necessary belongings for the hospital stay**

1. **How did you experience the preparation process before your surgery?**
   - Did you feel **secure, calm, fearful, worried, or stressed**?
   - How did these emotions affect your **motivation** (your willingness to follow instructions) for completing your preparations?
2. **Can you describe the support you relied on during your preparations for surgery?**
   - Knowledge of how to contact healthcare services for support.
   - Support from healthcare providers (information/dialogue) retrieved information, searching for information online, phone/email contact, mobile applications.
   - **Social support** (e.g., from family, friends, acquaintances, or neighbours)?
   - **Did you feel that any type of support was missing**

### **After your surgery at Home**

1. **Can you describe your experiences of communicating with healthcare services to obtain information after your surgery?**
   - How was the information provided? **Written information** from your clinic, v**erbal information** from your clinic phone call, healthcare visit, **E-health solutions**: video consultation, internet, mobile applications
   - To what extent did the information accommodate your personal needs and preferences?
   - How accessible, readable, and useful was the information for you?
   - What were the advantages and disadvantages for you personally?
2. **Can you describe how the information helped you perform self-care after your surgery?**

- Which aspects of self-care do you particularly remember?
- How easy or difficult was it for you to understand the information about self-care after surgery?
- What factors made your self-care easier or more challenging?
- Can you describe what you believe was expected of you in terms of self-care after surgery?

(If needed, remind the participant of common aspects of self-care after surgery, such as:)

- **Resting**
- **Maintaining appetite**
- **Taking regular medications**
- **Managing symptoms** (e.g., pain, nausea, constipation, dry mouth, appetite changes, fever)
- **Performing daily activities/exercises/training**
- **Maintaining bowel function**
- **Taking newly prescribed medications**
- **Assessing wound status and performing wound care, stoma care, or urinary catheter care**
- **Contacting healthcare services if complications arise**

1. **How did you experience managing your care at home after surgery?**

- Did you feel **secure, calm, fearful, worried, or stressed**?
- How did these emotions affect your **motivation** (your willingness) to perform self-care?

1. **Can you describe the support you used to perform self-care after your surgery?**

- **Knowledge of how to contact healthcare services for support.**
- **Support from healthcare providers (information/dialogue) retrieved information, searching for information online, phone/email contact, mobile applications.**
- **Social support (e.g., from family, friends, acquaintances, or neighbours)**
- **Did you feel that any type of support was missing**

Supplementary file 2 COMB

Matrix of the theoretical framework Capability, Opportunity and Motivation for Behaviour change (COM-B) and the Theoretical Domains Framework (TDF).

| **COM-B theme** | **TDF domain**  **Definition** | **Theoretical constructs represented in each domain** |
| --- | --- | --- |
| Psychological capability | Knowledge  (an awareness of the existence of something) | Knowledge (including knowledge of condition/scientific rationale; procedural knowledge; knowledge of task environment) |
|  | Cognitive skills  (A cognitive ability or proficiency acquired through practice) | Skills; skill development; competence; ability; interpersonal skills; practice; skill assessment |
|  | Memory  (The ability to retain information, focus selectively on aspects of the environment and choose between two or more alternatives) | Memory attention; attention control; decision-making; cognitive overload/tiredness. |
|  | Behavioural regulation  (Anything aimed at managing or changing objectively observed or measured actions) | Self-monitoring; breaking habit; action planning, anything aimed at managing or changing objectively observed or measured actions |
| Physical capability | Physical skills  (A physical ability or proficiency acquired through practice) | Skills; skill development; competence; ability; interpersonal skills; practice; skill assessment |
| Social opportunity | Social influences  (Those interpersonal processes that can cause individuals to change their thoughts, feelings, or behaviours) | Social pressure; social norms; group conformity; social comparisons; group norms; social support; power; intergroup conflict; alienation; group identify; modelling |
| Physical opportunity | Environmental context and resources  (Any circumstance of a person's situation or environment that discourages or encourages the development of skills and abilities, independence, social competence, and adaptive behaviour) | Environmental stressors; resources/material resources; organizational culture/climate; salient events/critical incidents; person x environment interaction; barriers and facilitators |
| Reflective motivation | Social/professional role and identity  (A coherent set of behaviours and displayed personal qualities of an individual in a social or work setting) | Professional identity; professional role; social identity; identity; professional boundaries; professional confidence; group identity; leadership; organizational commitment, a coherent set of behaviours and displayed qualities of an individual in a social or work setting |
|  | Beliefs about capabilities  (Acceptance of the truth, reality, or validity about an ability, talent, or facility that a person can put to constructive use) | Self-confidence; perceived competence; self-efficacy; perceived behavioural control; beliefs; self-esteem; empowerment; professional confidence |
|  | Optimism  (The confidence that things will happen for the best or that desired goals will be attained) | Optimism; pessimism; unrealistic optimism; identity |
|  | Intentions  (A conscious decision to perform a behaviour or a resolve to act in a certain way) | Stability of intentions; stages of change model; transtheoretical model and stages of change |
|  | Goals  (Mental representations of outcomes or end states that an individual wants to achieve) | Goals (distal/proximal); goal priority; goal/target setting; goals (autonomous/controlled); action planning; implementation intention |
| Automatic motivation | Reinforcement  (Increasing the probability of a response by arranging a dependent relationship, or contingency, between the response and a given stimulus) | Rewards (proximal, distal, valued/not valued, probable/improbable); incentives; punishment; consequences; reinforcement; contingencies; sanctions |
|  | Emotion  (A complex reaction pattern, involving experiential, behavioural, and physiological elements, by which the individual attempts to deal with a personally significant matter or event) | Fear; anxiety; affect; stress; depression; positive/negative affect; burn-out |

The relationship between the COMB themes and the TDF domains here are inspired from Atkins (2016). TDF domains are defined by Cane (2012).

Supplementary file 3 COMB

COREQ (COnsolidated criteria for REporting Qualitative research) Checklist

A checklist of items that should be included in reports of qualitative research. You must report the page number in your manuscript where you consider each of the items listed in this checklist. If you have not included this information, either revise your manuscript accordingly before submitting or note N/A.

| **Topic** | **Item No.** | **Guide Questions/Description** | **Reported on**  **Page No.** |
| --- | --- | --- | --- |
| **Domain 1: Research team**  **and reﬂexivity** | | | |
| *Personal characteristics* | | | |
| Interviewer/facilitator | 1 | Which author/s conducted the interview or focus group? | Title |
| Credentials | 2 | What were the researcher’s credentials? E.g. PhD, MD | Title |
| Occupation | 3 | What was their occupation at the time of the study? | 6 |
| Gender | 4 | Was the researcher male or female? | Title |
| Experience and training | 5 | What experience or training did the researcher have? | 6 |
| *Relationship with*  *participants* | | | |
| Relationship established | 6 | Was a relationship established prior to study commencement? | N/A |
| Participant knowledge of  the interviewer | 7 | What did the participants know about the researcher? e.g. personal  goals, reasons for doing the research |  |
|  |  |  | N/A |
|  |  |  |  |
| Interviewer characteristics | 8 | What characteristics were reported about the interviewer/facilitator?  e.g. Bias, assumptions, reasons and interests in the research topic |  |
|  |  |  | N/A |
|  |  |  |  |
| **Domain 2: Study design** | | | |
| *Theoretical framework* | | | |
| Methodological orientation and Theory | 9 | What methodological orientation was stated to underpin the study? e.g. grounded theory, discourse analysis, ethnography, phenomenology,  content analysis |  |
|  |  |  | 7 |
|  |  |  |  |
| *Participant selection* | | | |
| Sampling | 10 | How were participants selected? e.g. purposive, convenience,  consecutive, snowball |  |
|  |  |  | 5-6 |
|  |  |  |  |
| Method of approach | 11 | How were participants approached? e.g. face-to-face, telephone, mail,  email |  |
|  |  |  | 6 |
|  |  |  |  |
| Sample size | 12 | How many participants were in the study? | 10 |
| Non-participation | 13 | How many people refused to participate or dropped out? Reasons? | N/A |
| *Setting* | | | |
| Setting of data collection | 14 | Where was the data collected? e.g. home, clinic, workplace | 10 |
| Presence of non-  participants | 15 | Was anyone else present besides the participants and researchers? |  |
|  |  |  | 10 |
|  |  |  |  |
| Description of sample | 16 | What are the important characteristics of the sample? e.g. demographic  data, date |  |
|  |  |  | 10 |
|  |  |  |  |
| *Data collection* | | | |
| Interview guide | 17 | Were questions, prompts, guides provided by the authors? Was it pilot  tested? | 6 |
|  |  |  |  |
| Repeat interviews | 18 | Were repeat interviews carried out? If yes, how many? | N/A |
| Audio/visual recording | 19 | Did the research use audio or visual recording to collect the data? | 6 |
| Field notes | 20 | Were ﬁeld notes made during and/or after the interview or focus group? | N/A |
| Duration | 21 | What was the duration of the interviews or focus group? | 6 |
| Data saturation | 22 | Was data saturation discussed? | 8 |
| Transcripts returned | 23 | Were transcripts returned to participants for comment and/or | N/A |

| **Topic** | **Item No.** | **Guide Questions/Description** | **Reported on**  **Page No.** |
| --- | --- | --- | --- |
|  |  | correction? |  |
| **Domain 3: analysis and**  **ﬁndings** | | | |
| *Data analysis* | | | |
| Number of data coders | 24 | How many data coders coded the data? | 7 |
| Description of the coding  tree | 25 | Did authors provide a description of the coding tree? |  |
|  |  |  | 7 |
|  |  |  |  |
| Derivation of themes | 26 | Were themes identiﬁed in advance or derived from the data? | 7 |
| Software | 27 | What software, if applicable, was used to manage the data? | 7 |
| Participant checking | 28 | Did participants provide feedback on the ﬁndings? | N/A |
| *Reporting* | | | |
| Quotations presented | 29 | Were participant quotations presented to illustrate the themes/ﬁndings?  Was each quotation identiﬁed? e.g. participant number |  |
|  |  |  | 11-20 |
|  |  |  |  |
| Data and ﬁndings consistent | 30 | Was there consistency between the data presented and the ﬁndings? | 11-20 |
| Clarity of major themes | 31 | Were major themes clearly presented in the ﬁndings? | 11-20 |
| Clarity of minor themes | 32 | Is there a description of diverse cases or discussion of minor themes? | N/A |

Developed from: Tong A, Sainsbury P, Craig J. Consolidated criteria for reporting qualitative research (COREQ): a 32-item checklist for interviews and focus groups. *International Journal for Quality in Health Care*. 2007. Volume 19, Number 6: pp. 349 – 357
